# Sulfonyl fluorides as privileged warheads in chemical biology

**DOI:** 10.1039/c5sc00408j

**Published:** 2015-03-16

**Authors:** Arjun Narayanan, Lyn H. Jones

**Affiliations:** a Chemical Biology Group , BioTherapeutics Chemistry , WorldWide Medicinal Chemistry , Pfizer , 610 Main Street , Cambridge , MA 02139 , USA . Email: lyn.jones@pfizer.com

## Abstract

Sulfonyl fluoride electrophiles have found significant utility as reactive probes in chemical biology and molecular pharmacology. As warheads they possess the right balance of biocompatibility (including aqueous stability) and protein reactivity. Their functionality is privileged in this regard as they are known to modify not only reactive serines (resulting in their common use as protease inhibitors), but also context-specific threonine, lysine, tyrosine, cysteine and histidine residues. This review describes the application of sulfonyl fluoride probes across various areas of research and explores new approaches that could further enhance the chemical biology toolkit. We believe that sulfonyl fluoride probes will find greater utility in areas such as covalent enzyme inhibition, target identification and validation, and the mapping of enzyme binding sites, substrates and protein–protein interactions.

## Abstract 2

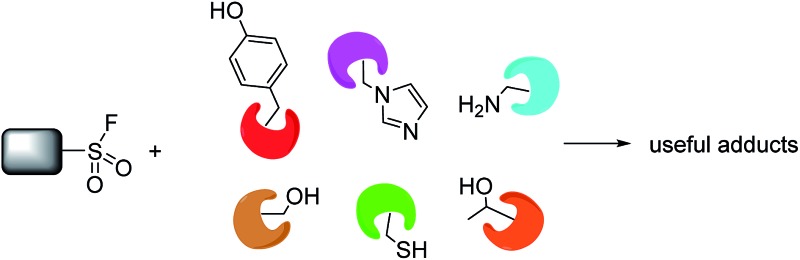
The use of sulfonyl fluoride probes in chemical biology is reviewed.

## Introduction

Covalent protein modifiers are extremely useful in the chemical biology arena, with uses including bioconjugation, activity-based protein profiling and target identification. The value of chemoproteomics and chemical probe development to drug discovery has also been emphasized, and new methods are needed to advance this field.^1,2^ There has been renewed interest in targeted covalent inhibitors from the drug discovery community,^3^ and this has also led to the development of companion chemical biology technologies that further pharmacological understanding, including target engagement determination in intact cells.^2^ This area has been dominated by the development of cysteine-reactive probes, which often apply acrylamide derivatives as a biocompatible functionality (to target kinase ATP-site cysteines for example).^4^ Another commonly used method to covalently modify target proteins is through the use of photoaffinity labelling, and this approach has found significant utility in target identification.^5^ However, the chemical biology toolkit still needs urgent attention to significantly improve and broaden the palette of useful synthetic transformations that can be harnessed to understand biology and drive drug discovery.

Sulfonyl fluorides (SFs) are privileged covalent warheads that can probe enzyme binding sites and assess functionally important protein residues. They possess desirable electrophilicity that enables capture of context specific amino acid reactivity whilst retaining appropriate aqueous stability commensurate with biomolecular experiments. Unlike sulfonyl chlorides, they are resistant to reduction since fluorine bond cleavage is exclusively heterolytic, and sulfonyl fluorides also possess significantly improved thermodynamic stability.^6^ In contrast to cysteine-targeted warheads such as acrylamides, SF electrophiles target active serine, threonine, tyrosine, lysine, cysteine and histidine residues.

This review highlights the development of SF probes in chemical biology and molecular pharmacology, and potential new applications will be presented. The review is divided into sections that describe the reactivity of sulfonyl fluorides with different amino acid residues.

## Serine (and threonine) reactivity

**Fig. 1:**
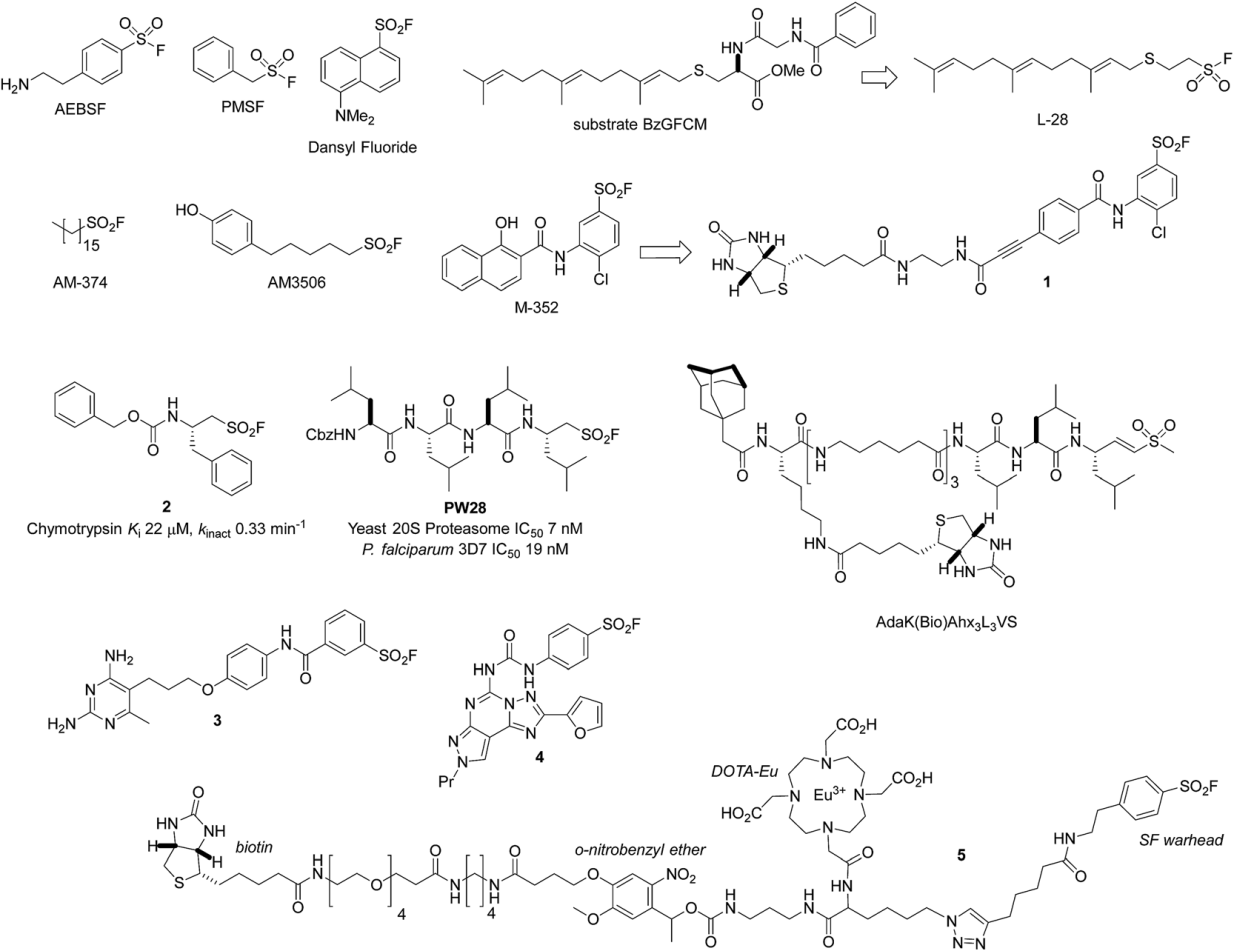
SF probes that react with serine: (2-aminoethyl)benzenesulfonyl fluoride (AEBSF); phenylmethylsulfonyl fluoride (PMSF); dansyl fluoride; L-28; AM-374 (palmityl sulfonyl fluoride); AM3506; **M-352**; **PW28**; enzyme inhibitors **1–5**; and cysteine-reactive probe AdaK(Bio)Ahx_3_L_3_VS.

Fahrney and Gold were the first to develop sulfonyl fluoride (SF) inhibitors of serine proteases.^7^ Among the commonly used SF reagents to inactivate these enzymes through reaction with the active site serine are (2-aminoethyl)benzenesulfonyl fluoride (AEBSF, the hydrochloride salt is called Pefabloc®), and phenylmethylsulfonyl fluoride (PMSF, Fig. 1). They are often used in the preparation of cell lysates to prevent the degradation of proteins of interest. Unsurprisingly, PMSF is significantly less soluble than AEBSF (it usually needs to be pre-dissolved in organic solvents before use), but more reactive, and it is rapidly degraded in aqueous solutions (half-life of 110 minutes at pH 7.5, 35 minutes at pH 8, 25 °C).^8,9^ As a result, AEBSF has found wider application as a serine protease inhibitor and is often prepared in a protease inhibitor cocktail for ease of use. Dansyl fluoride (Fig. 1) is another useful reagent as it labels serine proteases with a fluorescent group which can facilitate protein identification and enable fluorescence energy transfer studies.^10,11^ An extensive description of the use of AEBSF and PMSF to deactivate serine proteases is beyond the scope of this review, but we point the readers to a previous review of the area^12^ and the MEROPs database which is an excellent resource of proteolytic enzymes and commonly used inhibitors (; http://merops.sanger.ac.uk/inhibitors/index.shtml).^13^

Beyond the commercially available reagents AEBSF and PMSF, more bespoke SF probes and covalent inhibitors have been prepared to specifically target enzyme active site serines. SF inhibitors of the serine hydrolase polyisoprenylated methylated protein methyl esterase (PMPMEase) were developed to understand the relevance of this enzyme in cancer.^14^ The inhibitor L-28, based on the high affinity substrate BzGFCM, contains the farnesyl group which is accommodated in the hydrophobic binding site, was found to be significantly more potent than PMSF (IC_50_ 48 nM *versus* 1800 nM, Fig. 1). Similarly, efficient irreversible inhibition of lipoprotein lipase was reported using alkane sulfonyl fluorides of medium-to-long chain lengths (12 carbon atoms or more).^15^

Lipophilic SF inhibitors of fatty acid amide hydrolase (FAAH) were developed to irreversibly inhibit the enzyme, based on the previously described inhibitors PMSF and, in particular, AM-374 (palmityl sulfonyl fluoride, C16, Fig. 1).^16,17^ The potent inhibitor AM3506 (FAAH IC_50_ 5 nM) was used as a tool to explore the gastrointestinal, antihypertensive effects and stress reactivity of FAAH inhibition.^18–20^ Moreover, AM3506 was found to be a selective inhibitor when screened against a large number of serine hydrolases using activity-based protein profiling based on a rhodamine-tagged fluorophosphonate probe.^20,21^

**Fig. 2:**
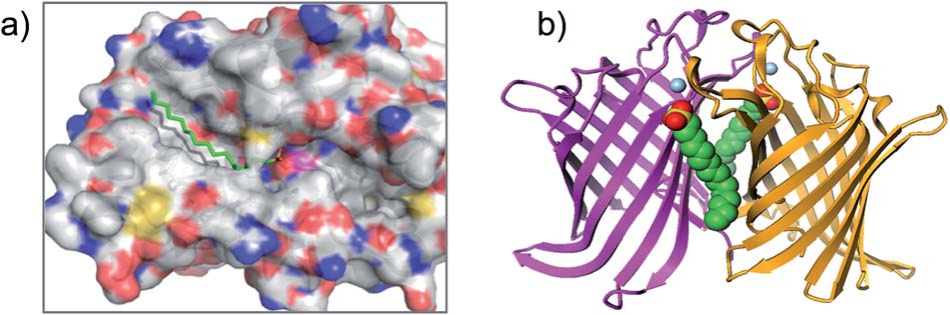
(a) X-ray crystal structure of the PPT1 and palmityl sulfonyl fluoride complex (PDB code 1EXW) highlighting the binding surface. (b) Crystal structure showing dimerisation of outer membrane phospholipase A (OMPLA) induced by palmityl sulfonyl fluoride (green, calcium ions blue spheres) (PDB code ; 1QD6).

The serine lipase palmitoyl-protein thioesterase-1 (PPT1) is involved in the catabolism of lipidated proteins during lysosomal degradation. Unlike related enzymes, PPT1 is insensitive to the commonly used reagent PMSF, though palmityl sulfonyl fluoride (AM-374) reacted covalently with the catalytic Ser115 as desired.^22^ A crystal structure of AM-374 with PPT1 showed how an aryl SF inhibitor could not be accommodated in the binding site, but the aliphatic palmityl tail was found to be located in a hydrophobic channel in the protein (Fig. 2a).

Palmityl sulfonyl fluoride has also been shown to induce dimerization of the outer membrane phospholipase A (OMPLA) enzyme through covalent modification of the active site serine residues.^23^ The substrate analogue increases stability of the dimer interface *via* hydrophobic interactions and recapitulates activation of the enzyme (Fig. 2b). A series of SF inhibitors bearing different chain lengths were then used to explore the thermodynamics of dimerization which showed that OMPLA is a finely tuned system that selects its appropriate phospholipid substrate in a specific manner.^24^

An activity-based probe of triacylglycerol lipases was created using a biotinylated SF inhibitor.^25^ Design of the probe **1** was based on the promiscuous and potent endothelial, lipoprotein and hepatic lipase inhibitor **M-352**, which enabled measurement of inhibitor enzyme engagement in a complex proteome, and an ELISA-based high throughput activity assay (Fig. 1).

More complex amino acid-derived aliphatic sulfonyl fluorides were developed as irreversible inhibitors of the serine protease chymotrypsin.^26^ A small number of derivatives were prepared to generate structure–activity relationships in a proof-of-concept study, resulting in the preparation of the optimal inhibitor **2** (Fig. 1). These molecules were also able to inhibit the cysteine protease papain (see cysteine reactivity section below). In a related piece of work, the same group also developed peptide sulfonyl fluoride (PSF) covalent proteasome inhibitors based upon known inhibitors such as bortezomib and epoxomicin.^27^ The most potent compound **PW28** possessed high selectivity for the β5 subunit of the proteasome and activity in a cell-based assay, albeit at higher concentrations (5 μM, Fig. 1). More importantly, subsequent research developed this initial concept into a novel approach for PSF inhibitors of the plasmodium proteasome. For example, **PW28** was also found to be an inhibitor of the *P. falciparum* proteasome that translated to potent antiparasitic activity. To determine the proteasome β-subunit selectivity of **PW28**, parasite extracts were pre-treated with the inhibitor, followed by the biotinylated vinyl sulfone promiscuous probe AdaK(Bio)Ahx_3_L_3_VS. **PW28** selectively inhibited the biotinylation of the β2 and β5 subunits by the probe, as determined by Western blot, reflecting its selectivity over the β1 subunit. **PW28** was also effective in suppressing *P. berghei in vivo*, and although it was found to be toxic in mice, the series appears significantly enabled for future optimization.^28^ More recent exploration of the mechanism of action of related PSF inhibitors of the immunoproteasome using mass spectrometry and structural analyses revealed a mode of action that involved Thr1 *O*-sulfonylation, followed by intramolecular displacement with K33 resulting in an inactivating cross-link.^29^

Rhomboid proteases are intramembrane serine proteases that possess important roles in biochemical signalling and are potential drug targets. A mass spectrometry assay was developed recently to discover covalent inhibitors of rhomboids with a view to creating activity-based probes.^30^ Through this screen, a PSF inhibitor was discovered, along with an isocoumarin irreversible inhibitor that was developed into an activity-based probe. These inhibitors not only serve as chemical leads for drug discovery, but also provide a means to determine target occupancy and subtype selectivity within the class.

For completeness, we searched the Protein Data Bank (PDB) for all X-ray crystal structures containing a ligand sulfone for which the sulfur atom was within 2.0 Å of any protein oxygen atom using Relibase.^31^ We additionally searched for sulfonate ester-modified serine residues. Upon manual curation of the resulting hits by visual inspection, and review of the primary citations, 38 PDB entries comprising 28 unique proteins (<95% pairwise sequence identity) were found to contain a sulfonate ester formed with a serine residue resulting from reaction with a sulfonyl fluoride containing ligand. These 28 proteins belong to six distinct PFAM clans and two isolated families (the subtilase family and the phospholipase A1 family), with 12 examples coming from the α/β-hydrolase superfamily. In all 28 cases, the modified serine was the residue serving as the nucleophile in the catalytic function of the enzyme.

Beyond serine hydrolases, a very early report from the Baker group described the irreversible inhibition of dihydrofolate reductase (DHFR) using the SF warhead.^32^ Diaminopyrimidines such as **3** (Fig. 1) were shown to inhibit mouse DHFR, possibly through serine modification, although this was never proven (see tyrosine reactivity section below).^33^ Similar approaches applied by the Baker group in the preparation of covalent SF inhibitors of guanine deaminase,^34^ xanthine oxidase^35^ and chymotrypsin^36^ were reported, although the reacting protein residue was not identified in these cases either.

Another example described the development of an irreversible A_3_ receptor bearing an aryl SF warhead (membranes containing the A_3_ receptor were pre-incubated with inhibitor **4**, washed and then shown not to bind a radioligand).^37^ Homology modelling based upon the rhodamine crystal structure and docking of **4** into the putative binding domain placed the SF moiety in close proximity to Ser247 which may be the reactive nucleophilic residue in this protein. These examples show that the incorporation of an SF warhead into a ligand template in the absence of protein structural information can be successful in creating covalent inhibitors. This technique could therefore be used in target identification strategies to deconvolute phenotypic screening hits, and complements photoaffinity approaches.

Another recent application of sulfonyl fluoride chemistry described the use of 4-fluorobenzene SF as a ^19^F probe of the enzyme subtilisin Carlsberg.^38^ Covalent inhibition through reaction with the active site Ser221 enabled an assessment of the local electronic environment and active-site motions using ^19^F NMR resonance.

The tetrafunctional molecule **5** (Fig. 1), containing an SF warhead to target serine proteases, was developed recently to enable protein quantification using an Eu-chelated DOTA to mediate inductively coupled mass spectrometry (ICP-MS).^39^ The probe also contained a biotin enrichment group and a photocleavable *o*-nitrobenzyl ether to integrate electrospray ionization MS ion trap MS (ESI-IT-MS) with the ICP-MS method of protein quantification. A preliminary study illustrated utility of the probe by enriching chymotrypsin from a mixture of 7 other non-SP proteins, and protein identification and quantification was effected using the integration of ICP-MS and ESI-IT-MS techniques. A previous study by the same group had used a simpler SF activity-based probe lacking the photolabile linker to detect serine proteases in a simple protein mixture.^40^ Optimization of probe and linker design, for example through the replacement of the biotin with a ‘silent’ click reporter (to mediate copper-mediated alkyne–azide cycloaddition, CuAAC),^41,42^ and DOTA alternatives, may enable intact cell-based activity, occupancy and imaging experiments in the future.

Seminal work by Koshland^43^ and Bender^44^ in 1966 demonstrated the first example of ‘chemical mutagenesis’ of a protein where a serine was converted to a cysteine residue in subtilisin. Treatment of the serine protease with PMSF converted the active site serine to a sulfonic ester that was displaced with thioacetate, and subsequently hydrolysed to the free thiol. In a similar manner, Koshland also showed that chymotrypsin, when treated with PMSF and then base, formed anhydrochymotrypsin (a serine-to-dehydroalanine conversion).^45,46^ This work showed that it was possible to convert serine–SF adducts into functionalized dehydroalanine derivatives that could undergo further chemistry.^47^

## Lysine reactivity

**Fig. 3:**
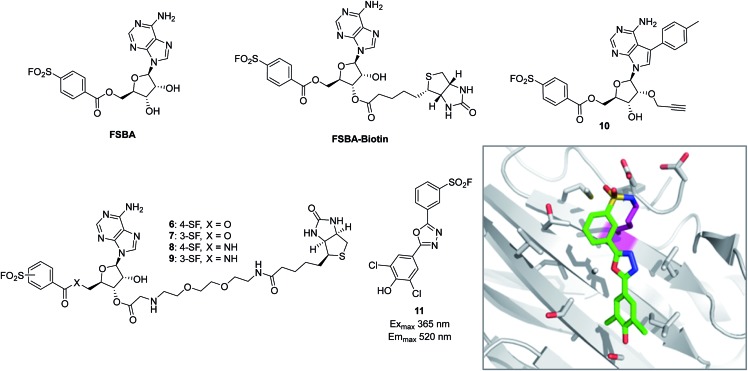
SF probes that react with lysine: FSBA; FSBA–biotin; **6**, **7**, **8**, **9**, **10**; structure of SF-containing oxadiazole **11** (emission and excitation maxima are quoted for the TTR-conjugate) and crystal structure showing adduct formation with TTR (PDB code ; 4FI7).

The reactive adenosine derivative 5′-fluorosulfonylbenzoyl 5′-adenosine (FSBA, Fig. 3) was originally developed 40 years ago by the Colman group to empirically explore nucleophilic residues in the binding sites of glutamate dehydrogenase.^48^ This ATP analogue became a commonly used covalent inhibitor and probe of ATP-binding proteins.^49^ FSBA contains an SF functionality that replaces the phosphoryl groups in ATP and peptide mapping has shown that the ε amino group of the conserved lysine in the ATP-binding site of kinases is the targeted nucleophilic residue.^50,51^ As a result, FSBA was used as an activity-based probe of recombinant kinases (ALK5 and CDK2) when used in conjunction with LCMS.^52^ Labelling of both kinases was inhibited by the promiscuous kinase inhibitor staurosporine, and ALK5 was selectively inhibited in a dose-related manner by the ALK5 selective inhibitor SB-431542. The same group later developed biotinylated FSBA derivatives (FSBA–biotin, Fig. 3) that enabled Western blot assessment of ALK5 inhibition by SB-431542.

A related approach was used recently to explore structure–function relationships in biotinylated FSBA derivatives and their ability to label recombinant kinases.^53^ The ester-linked probes **6** and **7** labelled recombinant LCK as shown by Western blot, but the amide derivatives **8** and **9** only weakly labelled the protein (Fig. 3). This could be due to the more restricted conformation of the amide derivatives preventing optimal placement of the SF functionality, as well as reducing the electrophilicity of the warhead. Interestingly, the *meta*-SF probe **7** appeared to be more selective for kinase labelling *versus* a small panel of non-kinase proteins, and was therefore used in further experiments to label additional kinases (p38 and FAK). Further characterization of **7** showed it to label LCK slowly (2 hours for optimal labelling) and high probe concentrations were required, suggesting further SAR is required to further optimize the technique.

In a more sophisticated approach, a clickable kinase inhibitor bearing the SF warhead was developed to label kinase targets in intact cells.^54^ Such probes enable more physiologically relevant target engagement to be assessed in-cell and avoid the disruptive nature of cell lysis where important biochemical information can be lost.^2,55^ The probe bears a *p*-tolyl group that is present in the Src inhibitor PP1 that occupies a hydrophobic pocket in this family of enzymes. Living cells were incubated with probe **10** (Fig. 3), lysed, conjugated with biotin-azide to enable streptavidin-mediated enrichment and western blotting, which confirmed the Src-family kinases as targets of the probe (Src, Yes, Lck, Blk, Lyn). Pre-treatment of cells with kinase inhibitors and subsequent competition of these kinases provided a way to determine in-cell kinase selectivity.

Beyond kinases, a small library of SF inhibitors of transthyretin (TTR) was developed to react covalently with the p*K*_a_-perturbed Lys15 in the thyroxine binding site.^56^ These inhibitors kinetically stabilized TTR thus preventing fibrillation (a cause of polyneuropathy). SF **11** (Fig. 3) reacted with TTR 1400 times faster than hydrolysis of this functionality, also illustrating the templated rate enhancement upon binding. Additionally, inhibitors containing the 2,5-diaryl-1,3,4-oxadiazole chromophore such as **11** were found to be fluorescent when conjugated to TTR, apparently relieving fluorescence quenching by SF, thus providing a method to develop environmentally sensitive turn-on probes.^57^ Probes for the nucleotide binding domains (NBD) of P-glycoprotein (P-gp) are useful in elucidating the mechanisms of drug transport across membranes related to multi-drug resistance (MDR). FSBA was used to study the ATP hydrolysis catalytic cycle and was shown to react in the ATP-binding site of P-gp using MALDI-TOF MS (Lys441 within the NBD1 region and Lys102 in NBD2 were modified).^58^

## Tyrosine reactivity

The chemistry of tyrosine is unique among the amino acid residues, and various methods have been developed to understand and harness the diversity of tyrosine reactivity, particularly in the field of chemical biology and bioconjugation.^59^

An early, and somewhat overlooked, report of the reaction of FSBA with a tyrosine residue is that of Esch and Allison, which described the use of ^14^C-labeled FSBA and tryptic digests to map sulfonylation to the nucleotide binding site of mitochondrial ATPase.^60^ There are several other reported examples of tyrosine labelling by FSBA, and often, nucleotide binding sites are labelled at lysine and tyrosine, so revealing important residues involved in catalysis.^49,61–65^ A recent example described affinity labelling of HCV replicase (NS5B) using FSBA followed by LC/MS/MS and MALDI-TOF peptide mapping that identified residues Tyr382 and Lys491 as major sites of modification.^66^ Mutation of these residues to alanine significantly reduced the RNA-dependent RNA polymerase activity of NS5B since Tyr382 is close to the suggested primer grip region and Lys491 is in the NTP channel.

To assess the nucleotide binding sites in cell lysate, a chemoproteomic approach was developed using FSBA as an activity-based probe.^67^ The technique was based on a peptide-centric approach called Combined Fractional Diagonal Chromatography (COFRADIC) which is able to sort labelled peptides based upon differences in retention properties in an HPLC run relative to parent (in this case facilitated by the cleavage of the labile benzoyl ester bond in FSBA adducts). MS//MS analysis of the sorted peptides identified 185 different labelled sites in Jurkat cell lysate, most of which occurred on tyrosine (67%) and to a lesser extent on lysine (33%) and serine (<1%). The proteins isolated included kinases, heat shock proteins, synthases, helicases and initiation, elongation and splicing factors. This work also suggested that when kinases were ‘affinity-loaded’ with FSBA, they were then able to label tyrosine in the protein substrate. For example, Lck was found to be labelled on Y393 which is the known autocatalytic phosphorylation site for the enzyme, and a Src substrate peptide could only be modified by FSBA in the presence of Src. These results suggest that SF probes can be used to map protein–protein interactions and identify enzyme substrates.

In a very elegant piece of work from the Colman group, the bifunctional affinity label 5′-((*p*-fluorosulfonyl)benzoyl)-8-azidoadenosine (5′-FSBAzA) was developed to react with bovine liver glutamate dehydrogenase in a two-step process (Fig. 7a).^68^ Following reaction of Tyr190 with 5′-FSBAzA in the dark, subsequent photolysis led to cross-linking with a region located near the C-terminus of the enzyme. This approach provided useful information regarding the proximity of protein residues and showed the compatibility of combining SF warheads with photoaffinity functionality, which could be applied to future chemoproteomic approaches.

The same group also used 4-(fluorosulfonyl)benzoic acid (FSB) to probe the nucleophilic residues in the active sites of several glutathione *S*-transferases in an unbiased manner.^69–71^ For example, using radiolabeled FSB and peptide mapping experiments, two tyrosine residues in pig lung glutathione *S*-transferase pi were found to react in a mutually exclusive manner to different degrees (69% Tyr7 and 31% Tyr106), which may be due to differences in phenol acidity (Tyr7 possesses a lower p*K*_a_ of 8.1 possibly due to the presence of a nearby arginine residue – see analysis below).^71^

**Fig. 4:**
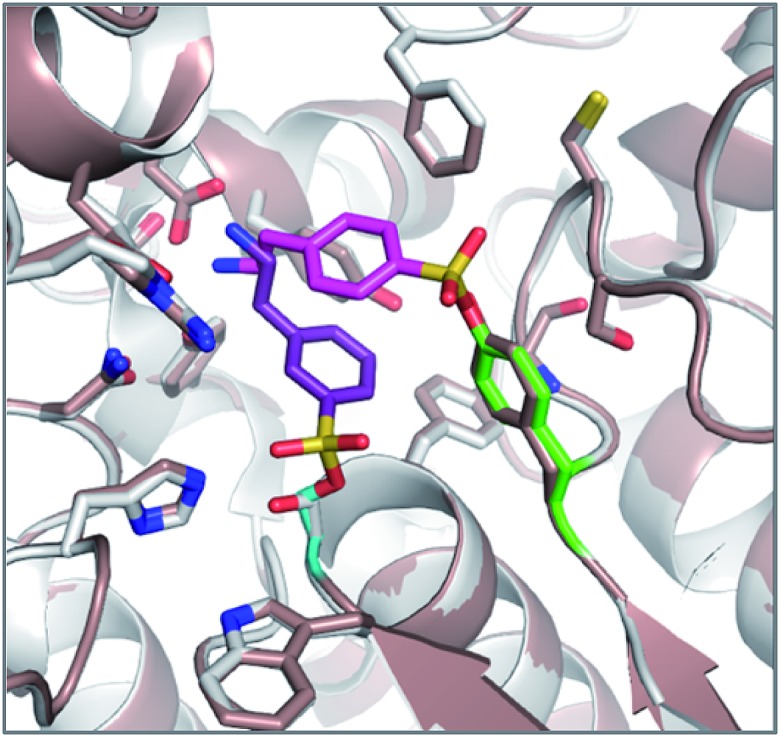
Structure of DPIV (grey) with AEBSF (magenta) adduct with Tyr547J (green). Overlaid is a model of DPIV (pink-brown) with a docked *meta*-analogue of AEBSF (purple) after reaction with Ser630 (cyan). Covalent docking was performed using the Covalent Docking module from the Schrodinger 2014-3 suite (Schrodinger, LLC).

The reagents AEBSF and PMSF described in the section above had also been found to react unexpectedly with tyrosine residues previously,^72^ and there is a report of AEBSF even reacting with tyrosine within a serine protease active site.^73^ In this specific case, Tyr547 in dipeptidyl peptidase IV (DPIV) was modified in preference to the active site Ser630. A crystal structure of the complex showed that the protonated amine group in AEBSF interacted with residue E205 as seen for the ammonium group of P2 peptide substrates, and the *para*-disposition of the SF group enabled reaction with the flexible Tyr547 residue (Fig. 4). It would be interesting to see if the *meta*-SF derivative of AEBSF would react with the catalytic serine as this would appear to be possible from the docked structure as shown in Fig. 4.

**Fig. 5:**
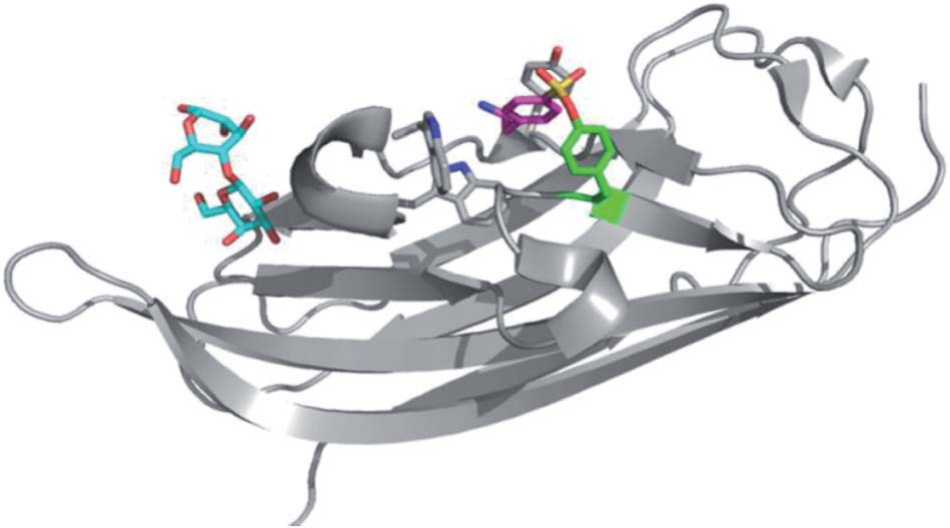
AEBSF (magenta) adduct of dscPsaA in complex with galactose (cyan) (PDB 4F8O).

The pH 6 antigen (Psa) of *Yersinia pestis* is a key virulence factor critical to plague pathogenesis. To elucidate the structural basis for Psa binding to its receptors, the protein was cocrystallized with galactose (found in glycosphingolipids), and interestingly the complex had formed an adduct with AEBSF which was used as a reagent during protein purification (Fig. 5).^74^ AEBSF had reacted with a tyrosine residue (Y100) in a hydrophobic depression near the sugar binding niche, which is the phosphatidylcholine (PC) site in Psa (responsible for additional interactions with alveolar epithelial cells). Y100 is essential for mediating an ‘aromatic guidance’ mechanism, where cation–π interactions are formed with PC. In another instance of coincidental labelling of tyrosine by AEBSF, a crystal structure of the RNA bacteriophage φ6 lysin revealed modification of Tyr196 (PDB ; 4DQJ).^75^ These and other examples^72^ show that SF adducts are formed regularly when covalent ‘fragment’ inhibitors such as AEBSF and PMSF are used as additives during protein isolation and purification procedures to reduce protease activity. This becomes particularly important when performing experiments such as activity-based protein profiling on cell lysate, since target proteins of interest could be modulated by SF reaction (AEBSF for example is often used at concentrations 0.1–1 mM), suggesting that it is preferable to develop such techniques using intact cells.

**Fig. 6:**
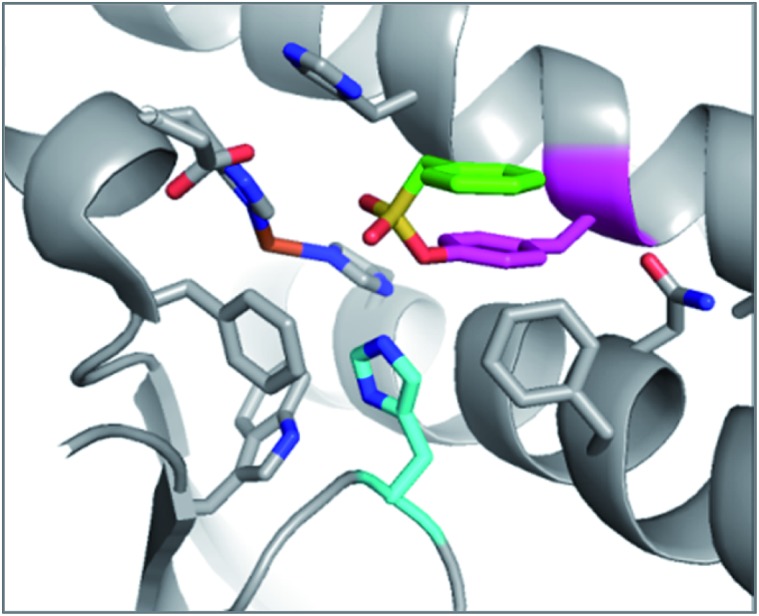
X-ray structure of PMSF (green) that has reacted with Tyr41 (magenta) in Fe-SOD in *Sulfolobus solfataricus* (PDB ; 1WB8). The proximal His155 (cyan) and Fe^III^ (brown) are also highlighted.

PMSF was found to react with the functionally important tyrosine residue Tyr41 in the active site of iron superoxide dismutase (Fe-SOD) from the hyperthermophile *Sulfolobus solfataricus*.^76^ His155 forms a hydrogen bond with the modified Tyr41 oxygen, potentially stabilizing anion formation and increasing nucleophilicity of Tyr41 (Fig. 6). This result suggests that SF activity-based probes could be developed for this enzyme class, especially as many of the conserved tyrosines are adjacent to basic histidine residues in their active sites (see analysis below).

**Fig. 7:**
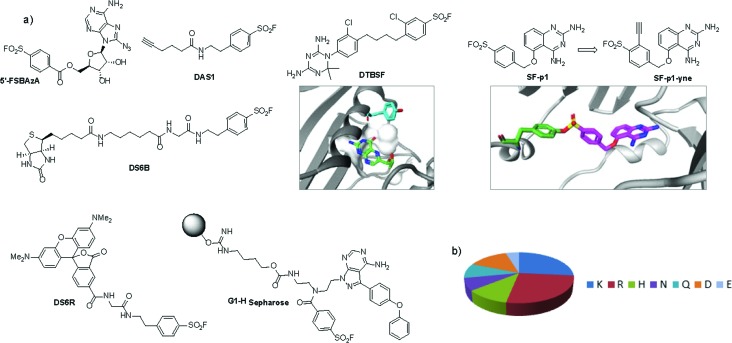
(a) SF probes that react with tyrosine: 5′-FSBAzA; DAS1; DS6B; DS6R; G1-H sepharose resin; DTBSF and complex of biopterin (green) in chicken liver DHFR (PDB 1DR1) showing proximity of Tyr31 (cyan) which reacts with DTBSF; **SF-p1** and clickable probe **SF-p1-yne** (and crystal structure of **SF-p1** with DcpS, PDB ; 4QDV). (b) Pie chart showing the distribution of amino acid residues proximal to known SF-reactive tyrosines as determined by analyzing crystal structures in the PDB.^90^

An unbiased chemoproteomic study using clickable, biotinylated and rhodamine reporter-tagged AEBSF (DAS1, DS6B and DS6R respectively, Fig. 7) confirmed labelling of functionally important tyrosines in GST enzymes in complex proteomes.^77^ A number of other proteins were labelled by DAS1 on Tyr, including acetyl-CoA acetyltransferases, amylase 2, carboxypeptidase B1, elongation factor 2, glycine *N*-methyltransferase, beta-globin, heat shock cognate protein Hspa8 and threonyl-tRNA synthetase, although the relevance of binding at many of these sites has yet to be determined. An attractive application of this observation was described recently to enable a new method for GST fusion protein immobilization.^78^ In a proof-of-principle experiment, ITK and EGFR kinases were fused to GST, immobilized onto sepharose beads bearing the SF probe (G1-H sepharose, Fig. 7a), and their activities were shown to be retained using a simple enzyme assay.

Interestingly, relative to the previous report of alluding to potential serine targeting of the SF diaminopyrimidine DHFR inhibitor **3**, a related covalent inhibitor, DTBSF, was actually found to fortuitously modify residue Tyr31 in chicken liver DHFR (Fig. 7).^79^ This can be explained by the likely proximity of this residue to the SF functionality, as shown in a crystal structure of biopterin (which binds in the same site) with DHFR (Fig. 7a).^80^ *p*-Nitrobenzenesulfonyl fluoride (NBSF) is a highly reactive electrophilic reagent described as a tyrosine-specific reagent to map reactive and functionally important tyrosine residues. Examples of its use in this manner include DNase,^81^ estradiol 17β-dehydrogenase,^82^ glucocorticoid receptor,^83^ the placental taurine transporter and H^+^ pump,^84,85^ histones,^86^ dopamine receptors,^87^ avidin and streptavidin,^88^ and snake venom phospholipases,^89^ although there are no recent examples of its use in the literature, presumably due to the development of less reactive and more selective reagents as described above.

Our own efforts in this area resulted in the first examples of deliberately targeting enzyme binding site tyrosine residues using SF probes.^90^ Decapping scavenger enzyme (DcpS) is responsible for removing the m^7^-GTP cap from the 5′-end of mRNA fragments, and it is also involved in microRNA metabolism. DcpS was originally identified as the target of survival motor neuron (SMN) modulators for the treatment of spinal muscular atrophy that were found in a phenotypic screen. To validate the target, a new method was required to measure target occupancy of the enzyme in intact cells, and therefore a clickable covalent probe was required. The binding site does not contain cysteine, but several tyrosine residues that were targeted specifically by different SF regioisomeric probes (for example, probe **SF-p1**, Fig. 7). An alkyne-tagged SF inhibitor (**SF-p1-yne**) was able to measure DcpS target engagement in cells previously treated with an inhibitor. Interestingly, a proximal lysine residue in the binding site did not react with the SF chemical probes, which suggested that the microenvironment of the protein is playing an important role in determining residue reactivity. As a result, a review of the literature, the PDB and the proteomics experiments carried out in Hanoulle *et al.* ^67^ and Gu *et al.* ^77^ was performed to assess residues proximal to SF-reactive tyrosine residues. As can be seen in Fig. 7b, the majority of residues proximal to reactive tyrosines are basic in nature, whilst acidic and some neutral residues are also represented. In nearly all cases, a proximal residue facilitates the deprotonation and reactivity of the tyrosine –OH functionality. This analysis will help delineate the structure–reactivity relationships of amino acid residues with electrophilic warheads.

## Other residues

Histidine and cysteine also react with sulfonyl fluorides, although these adducts appear to be unstable relative to those described above. FSBA was shown to react with Tyr368 in the active site of bovine mitochondrial F_1_-ATPase at pH 8.0 that switched to His427 at pH 6.0 (both residues were modified at pH 7.0).^91^ In another example, His130 was inferred as the target of FSBA in *Salmonella typhimurium* 5-phosphoribosyl-α-1-pyrophosphate synthetase due to the identity of the labelled peptide sequence following trypsin digestion and its labile nature.^92^

Although unstable, such reversible covalent interactions could be useful in the creation of potent protein binders and chemical biology tools that target His and Cys residues. Cysteine cross-linking, mediated *via* thiosulfonate formation, could be harnessed to deliberately inactivate protein targets, in a manner similar to other modes of cysteine redox switching. For example, it was postulated that an ATP-site cysteine in rabbit muscle pyruvate kinase reacts with FSBA to form a thiosulfonate intermediate that is subsequently quenched by an additional neighbouring cysteine, thus forming a partially active disulfide enzyme.^93^ Similarly, isocitrate dehydrogenase reacted with FSBA to form a disulfide bond that inhibited ATPase activity.^94^

**Fig. 8:**
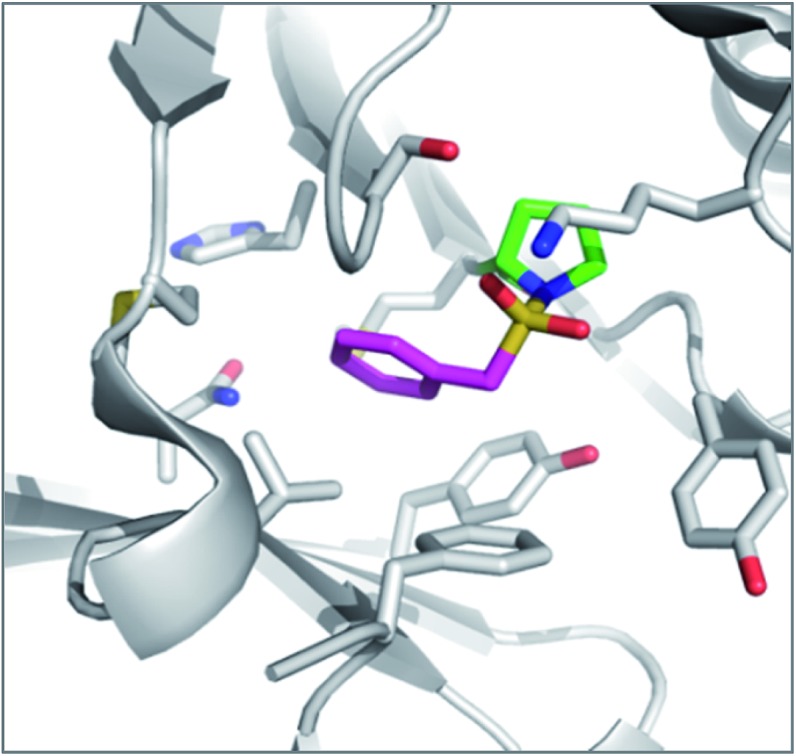
X-ray structure of PMSF (magenta) with macrophage migration inhibitory factor (MIF) illustrating the reaction with the N-terminal proline residue (green). PDB code 3CE4.

PMSF was found to react with the N-terminal proline amine group in macrophage migration inhibitory factor (MIF) unexpectedly (Fig. 8).^95^ Pro1 is the catalytic residue in the tautomerase active site of the enzyme and its lower p*K*_a_ presumably facilitates SF reactivity. Additionally, the flexibility afforded by the methylene link in PMSF enables the benzene ring to be optimally positioned within the hydrophobic cavity.

## Conclusions

Much of the reactivity of SF reagents with amino acid residues in proteins was discovered serendipitously. Nevertheless, they have become important chemical biology and pharmacology tools that enable the targeting of context-specific Ser, Thr, Tyr, Cys, Lys and His residues. The examples highlighted in this review support the application of SF probes in chemoproteomic experiments due to their biocompatible and privileged reactivity. A combination of the selectivity of binding site-templated reactivity and stabilization of the fluoride leaving group in aqueous systems provides the SF functionality with unique properties for incorporation into activity-based probes.^6^ However, caution should be exercised when using fragment-like SF inhibitors due to their somewhat intrinsic promiscuity. For example, the protease inhibitors AEBSF and PMSF are regularly used in cell lysate experiments at high concentrations to prevent degradation of the protein of interest, but they are known to react with a number of other proteins. This is another reason why best efforts should be made to develop chemoproteomic approaches on intact cells, to more accurately sample physiologically-relevant biochemistry and pharmacology.

The examples shown in this review highlight opportunities for the rational design of SF activity-based probes to provide optimal coverage of the reactive proteome. Computational techniques can also be used to better understand and then predict the reactivity of amino acid residues in proteins (trained on the results of such chemoproteomic experiments), and influenced by the environment of the binding pocket that can perturb various physicochemical parameters such as p*K*_a_. Further technical enhancements in this area could be realized through the use of high quality antibodies that recognize sulfonyl-tagged amino acids to enable specific enrichments. For example, antibodies generated to an FSB–KLH immunogen were generated previously to enable identification of FSBA-labelled proteins from lymphoid cells.^96^ Similarly, antibodies to FSBA-labelled kinases were generated previously and used to immunoprecipitate PKC-β_1_ (ref. 97) and anti-FSBA antibodies are now commercially available.

The development of SF chemistry will broaden the toolkit of useful synthetic transformations in chemical biology. Additionally, the ease with which sulfonyl fluorides can be prepared (including the commercial availability of SF-containing synthetic monomers for pendant functionalization), their compatibility with click functionalities such as azides and terminal alkynes, and their chemical stability means these electrophilic warheads will likely find even greater utility in the future.^6^

There are some reports of using SF probes to map enzyme binding sites and substrates, and therefore target identification would appear to be another suitable application of SF chemistry, may be as an alternative to photoaffinity labelling. Since certain SF adducts can be hydrolysed, they may also find utility in developing traceless linkers to PEG and other bioconjugates that are often used to modulate the pharmacodynamics and pharmacokinetics of protein therapeutics.
